# Clinical effectiveness of cefiderocol for the treatment of bloodstream infections due to carbapenem-resistant *Acinetobacter baumannii* during the COVID-19 era: a single center, observational study

**DOI:** 10.1007/s10096-024-04833-8

**Published:** 2024-04-18

**Authors:** Alessandra Oliva, L Liguori, S Covino, F Petrucci, F Cogliati-Dezza, A Curtolo, G Savelloni, M Comi, F Sacco, G Ceccarelli, A Viscido, F Alessandri, G Raponi, F Pugliese, CM Mastroianni, M Venditti

**Affiliations:** 1 Department of Public Health and Infectious Diseases, Sapienza University of Rome, Piazzale Aldo Moro 5, Rome, 00185 Italy; 2 Microbiology and Virology Laboratory, Department of Molecular Medicine, Sapienza University of Rome, Rome, Italy; 3 Department of General and Specialistic Surgery, Sapienza University of Rome, Rome, Italy

**Keywords:** Cefiderocol, Carbapenem-resistant *Acinetobacter baumannii*, Bloodstream infection, Ventilator-acquired pneumonia, Pneumonia, Colistin

## Abstract

**Background:**

We assessed the clinical effectiveness of cefiderocol (CFDC) in comparison with colistin (COL) for the treatment of carbapenem-resistant *Acinetobacter baumannii* (CRAB) bloodstream infections (BSI).

**Materials/methods:**

Retrospective cohort study including adults with CRAB-BSI. Outcomes were mortality, clinical cure and adverse events during therapy. The average treatment effect of CFDC compared to COL was weighted with the inverse-probability treatment weight (IPTW).

**Results:**

Overall, 104 patients were included (50 CFDC, 54 COL), median age 66.5 years, median Charlson Comorbidity Index 5, septic shock in 33.6% of patients. Primary BSI accounted for 43.3% of cases, followed by ventilator-associated pneumonia (VAP) (26%), catheter-related BSI (20.2%) and hospital-acquired pneumonia (HAP) (9.6%). Although not significantly, mortality at all time points was lower for CFDC than COL, while clinical cure was higher in CFDC than COL (66% vs. 44.4%, *p* = 0.027). Adverse events were more frequent in COL than CFDC-group (38.8% vs. 10%, *p* < 0.0001), primarily attributed to acute kidney injury (AKI) in the COL group. Patients with bacteremic HAP/VAP treated with CFDC had a significant lower 30-d mortality and higher clinical cure than COL (*p* = 0.008 and *p* = 0.0008, respectively). Increment of CCI (*p* = 0.005), ICU (*p* = 0.025), SARS-CoV2 (*p* = 0.006) and ECMO (*p* < 0.0001) were independently associated with 30-d mortality, while receiving CFDC was not associated with survival.

**Conclusions:**

CFDC could represent an effective and safe treatment option for CRAB BSI, especially in patients with bacteremic HAP/VAP and frail patients where the risk of acute renal failure during therapy should be avoided.

**Supplementary Information:**

The online version contains supplementary material available at 10.1007/s10096-024-04833-8.

## Key summary points

Increasing real-life data support the clinical effectiveness and safety of cefiderocol (CFDC) for carbapenem resistant *Acinetobacter baumannii* (CRAB) infections.

We investigated CFDC in comparison with colistin (COL) for the treatment of CRAB bloodstream infections (BSI).

Clinical cure was higher in CFDC than COL group.

Patients with hospital acquired/ventilator-associated pneumonia treated with CFDC had a statistically significant lower 30-d mortality and higher clinical cure than those treated with COL.

Adverse events were more frequent in COL than in CFDC-group.

CFDC could be an effective and safe treatment option for CRAB BSI, especially in patients with HAP/VAP and frail patients where the risk of acute renal failure during therapy should be avoided.

**Supplementary Information:**

The online version contains supplementary material available at 10.1007/s10096-024-04833-8.

## Introduction

Antibiotic resistance is a major global health concern impacting both medical costs and mortality rates [1]. In particular, resistance to carbapenems in *Acinetobacter baumannii* exceeds 70% in Southern and Eastern European regions, including Italy [2–4].

Carbapenem-resistant *A. baumannii* (CRAB) poses a significant threat, particularly in Intensive Care Unit (ICU) settings [5]. Mortality rates are high, ranging from 50 to 70%, especially in cases of septic shock and ventilator-associated pneumonia (VAP) [4].

While colistin is considered a mainstay in CRAB treatment, mostly in combination with other antibiotics [6], its use is limited by poor lung penetration [7] and risk of nephrotoxicity [7–10]. Furthermore, many strains of CRAB have recently developed resistance against colistin and other polymyxins [4, 11].

In this context, cefiderocol (CFDC), a new siderophore cephalosporin approved by the Food and Drug Administration for the treatment of serious infections caused by carbapenem-resistant Gram-negative bacteria (CR-GNB) [12], might offer a compatible alternative in the treatment of CRAB infections.

While the phase 3 trial CREDIBLE-CR revealed that, within the subset of CRAB infections, patients treated with CFDC experienced higher mortality rates compared to those receiving the best available therapy [13], subsequent real-life studies have shown promising outcomes regarding improved clinical efficacy and safety with CFDC, particularly in the context of bloodstream infections (BSI) and ventilator-associated pneumonia (VAP) [14–22]. Consequently, the debate concerning the role of CFDC in treating CRAB infections remains ongoing and necessitates additional evidence from real-world settings.

The aim of the study was to evaluate the clinical effectiveness and safety of CFDC in comparison with colistin (COL) for the treatment of CRAB BSI during the COVID-19 era.

## Materials and methods

### Study design

This is an observational, retrospective, single-centre study including adult patients diagnosed with CRAB BSI and hospitalized at a large Academic Hospital between June 2021 and April 2023.

Patients were categorized into those treated with CFDC- or COL-based regimens.

Inclusion criteria were (i) age > 18 years, (ii) hospitalization for at least 48 h and (iii) receipt of CFDC or COL as the definite treatment for CRAB BSI. Patients aged < 18 years, those receiving agents other than CFDC or COL or those deceased before the blood culture (BC) results had been available were excluded.

### Variables

Collected data included age, gender, ward of index BC (Intensive Care Unit, ICU, or non-ICU), date of admission, previous antibiotic therapy, previous CRAB colonization, days of hospitalisation prior to infection, presence of indwelling central venous catheters, comorbidities such diabetes mellitus, cardiovascular, renal failure or haemodialysis, solid and haematological malignancies, cirrhosis, hepatopathy, obesity (defined as BMI≥30), immunosuppression, chronic obstructive pulmonary disease (COPD), HIV infection. Concomitant SARS-CoV2 infection was also recorded. Burden of comorbidities was assessed by means of Charlson Comorbidity Index (CCI) [23] while for severity at ICU admission we calculated the Simplified Acute Physiology Score (SAPS). Variables related to the infection included source of BSI, presence of septic shock at infection onset, mechanical ventilation and/or need of continuous renal replacement therapies (CRRT), extra-corporeal membrane oxygenation (ECMO), laboratory parameters such as C-reactive protein (CRP) and procalcitonin (PCT). Empiric and definitive antibiotic regimens, as well as their use in monotherapy or in combination, were also collected.

### Antimicrobial treatment decision and appropriateness of therapy

The choice of antibiotic therapy was at discretion of the treating Infectious Diseases consultants. Intravenous antibiotics were administered as follows: CFDC 2 gr loading dose followed by a 3 h infusion of 2 g every 8 h; COL 9 MUI loading dose followed by 4.5 MUI every 12 h; ampicillin/sulbactam (A/S) with a total daily dose of 24–27 g (4 g/2 g every 6 h or 6 g/3 g every 8 h); fosfomycin (FOF) with a total daily dose of 16–18 g, divided every 6–8 h; tigecycline, first dose of 100–200 mg, followed by 50–100 mg every 12 h; meropenem 2 g loading dose followed by 2 g every 8 h. Dose adjustments for all antibiotics were made based on renal function, following the manufacturer’s recommendations.

Early (< 24 h) appropriate antibiotic therapy was reached when at least one drug started within 24 h from the collection of index BC was subsequently found to be active in vitro.

Definitive appropriate therapy was defined if the isolated CRAB was found to be susceptible at least to one antibiotic of the final treatment and if it was started within 48 h from BSI onset.

### Study outcomes

Primary outcomes were all-cause mortality at 7, 14 and 30-d following BSI onset, while secondary outcomes were clinical and microbiological cure, occurrence of adverse events during treatment, CRAB infection recurrence and 30-d superinfections.

### Definitions

Immunosuppression was defined as use of prednisolone (or equivalent) > 0.5 mg/kg/day for > 1 month, chemotherapy or immunotherapy in the last 3 months.

Infections were defined according to the CDC/NHSN criteria [24]. Hospital acquired/ventilator-associated pneumonia (HAP/VAP) were defined in accordance with CDC/NHSN surveillance definition of healthcare-associated infection for pneumonia with specific criteria [25]. VAP was defined as pneumonia in patients who had a device to assist or control respiration continuously through a tracheostomy or by endotracheal intubation within the 48 h period before the onset of infection. Primary BSI was defined as BSI occurring in patients without a recognized source of infection. Catheter-related BSI (CR-BSI) was defined if the semiquantitative culture of the catheter tip was positive for the same CRAB isolated from the blood [26]. The likely or ascertained source of BSI was indicated by the attending physician or by the Infectious Disease consultants (AO, GC) in the medical record according to guidelines [27]. In case of doubt, a panel discussion was performed.

The indicative parameters of early (48–72 h) clinical improvement were at least one of the following: discontinuation of treatment with inotropic drugs if the patient was previously in septic shock, disappearance of fever for at least 48 consecutive hours after the start of treatment, reduction of serum procalcitonin values by at least 80% compared to the initial value or achievement of a serum PCT value < 0. 5 ng/mL, a reduction of at least 75% of the maximum achieved value of c-reactive protein (PCR) [28].

Clinical cure was defined as the resolution of symptoms after the end of antibiotic treatment. Microbiological cure was defined as negative follow-up BCs with eradication of CRAB from the start of definitive therapy. CRAB infection recurrence was defined as a new isolation of the same CRAB from BC or other sites within 30 days after the clinical recovery. Superinfections were recorded at 30 days following the start of definitive treatment. Acute kidney injury (AKI) was defined as an increase in serum creatinine by ≥ 0.3 mg/dl (≥ 26.5 µmol/l) within 48 h or an increase in serum creatinine to ≥ 1.5 times baseline from the start of antibiotic therapy [29, 30].

### Microbiology

According to the hospital microbiology laboratory routines, bacterial pellet obtained from positive BCs was used for bacterial identification by the Matrix-Assisted Laser Desorption Ionization–Time Of Flight Mass Spectrometry (MALDI-TOF MS) system (Bruker Daltonik GmbH, Bremen, Germany). Isolated colonies from other biological samples (sputum or lower respiratory samples in cases of HAP/VAP as source of BSI, catheter’s tip in case of CR-BSI or wound/abscesses in cases of skin and soft tissue as source of BSI) were also identified by MALDI-TOF MS system.

Antimicrobial susceptibility testing was performed with the Vitek 2 automated system (bioMérieux, Marcy l’Etoile, France) and Microscan Walkaway (Beckman and Coulter, Brea, California, USA) system. For CFDC susceptibility, the disk diffusion method was used, and the diameter of inhibition was calculated and interpreted in accordance with guidelines [31]. In instances where we provided the precise value of CFDC Minimum Inhibitory Concentration (MIC), the ComASP® Cefiderocol (Liofilchem, Roseto degli Abruzzi, Italy) was utilized, following the manufacturer’s instructions.

### Statistical analyses

Categorical variables were described through absolute frequencies and percentages; quantitative variables were reported through median with interquartile range or mean and SD, depending on the normal or non-normal distribution of the data. Differences between qualitative variables were analysed by means of Chi-square or Fischer tests, while differences between quantitative variables were assessed by means of t-Student or Mann-Whitney tests, as appropriate. Multivariate Cox regression model was performed to sort out the independent predictors of mortality within 30 days from BSI onset, accounting for covariables.

The average treatment effect of CFDC compared to COL was weighted using the inverse-probability treatment weight (IPTW) accounting for variables potentially influencing the treatment (SARS-CoV2 infection, CKD, tumor, septic shock) and the outcome, such as ICU, SARS-CoV2, septic shock, CRRT, ECMO, source of infection (VAP vs. other), diabetes mellitus, age and burden of comorbidities.

To assess the balance among the variables, we calculated the standardized mean difference before and after the IPTW procedure for each variable that could potentially influence the treatment.

*P*-value analyses were two-sided and a *p*-value of less than 0.05 was considered statistically significant. All statistical analyses were performed using STATA™ software, v. 17 (StataCorp) and Graphpad Prism™, charts using Microsoft Office™ and Graphpad Prism™.

The study was conducted in accordance with the principles of the Declaration of Helsinki. The protocol was approved by the local Ethics Committee. The clinical and diagnostic management of the patients was already carried out according to normal clinical practice. Informed consent was waived due to the retrospective nature of the research.

## Results

### Study population

During the study period, 104 patients satisfied our inclusion criteria. Among them, 50 (48.1%) patients were treated with CFDC and 54 (51.9%) with COL. The median age was 66.5 (IQR 58–78) years, 71 (68.3%) patients were male and the median CCI was 5 (IQR 2–7), with a slightly higher CCI in CFDC than COL. At the time of infection onset, 44 (42.3%) patients had a SARS-CoV2 infection, more commonly observed in the COL group (53.7% vs. 30%, *p* = 0.015) and 66 (63.6%) were hospitalized in the ICU (64.8% vs. 62% in the CFDC and COL group respectively, *p* = 0.766). Mechanical ventilation was present in 51 (49.5%) patients and 5 (4.8%) patients needed ECMO at the time of infection. Septic shock was present in 33.6% of subjects, higher in the COL group (42.6% vs. 24%). The most frequent source of BSI was primary BSI (45, 43.3%), followed by VAP (27, 26%), CR-BSI (21, 20.2%) and HAP (10, 9.6%), with no significant differences observed between the groups.

Overall, polymicrobial BSIs were identified in 23 out of 104 patients (22.1%), distributed as follows: 6 cases of CRAB/*E. faecalis*, 5 cases of CRAB/KPC-producing *K. pneumoniae*, 5 cases of CRAB/vancomycin-resistant *E. faecium* (VRE), 2 cases of CRAB/*S. aureus*, 2 cases of CRAB/*Candida* spp, 2 cases of CRAB/*E. cloacae*, 1 case of CRAB/KPC-producing *K. pneumoniae*/*Candida spp*.

Specifically, among the patients with lung infections, 7 cases exhibited polymicrobial BSI, distributed as follows: 2 cases of CRAB/*E. faecalis*, 2 cases of CRAB/VRE, 1 case of CRAB/*Candida spp*, 1 case of CRAB/KPC, 1 case of CRAB/*E. cloacae*. Notably, in only the latter two cases, KPC and *E. cloacae* were also detected in bronchoalveolar lavage (BAL) samples, suggesting a genuine polymicrobial lung infection.

The full baseline demographic and clinical features of study population are shown in Table 1.

**Table 1:** General features and outcomes of study population

	Overall population n (%) = 104 (100)	CFDC n (%) = 50 (48.1)	COL n (%) = 54 (51.9)	*p*-value
Male sex, n (%)	71 (68.3)	32 (64)	39 (72.2)	*0.368*
Age, median (IQR), years	66.5 (58–78)	69 (58–77)	64 (58–78)	*0.580*
Hospital length of stay before infection onset, median (IQR), days	21.5 (12.5–39.5)	24 (13-44.5)	19 (11.5–31.5)	*0.24*
Previous (90 days) hospitalization, n (%)	80 (76.9)	36 (72)	44 (81.5)	*0.252*
Previous (90 days) antibiotic treatment, n (%)	76 (73.1)	35 (70)	41 (75.9)	*0.496*
ICU stay, n (%)	66 (63.5)	31 (64.8)	35 (62)	*0.766*
SARS-CoV-2 co-infection, n (%)	44 (42.3)	15 (30)	29 (53.7)	***0.015***
Charlson Comorbidity Index, median (IQR)	5 (2–7)	5 (3–7)	4.5 (2–6)	*0.409*
CCI ≥ 3, n (%)	76 (73.1)	40 (80)	36 (66.7)	*0.126*
Diabetes, n (%)	23 (22.1)	9 (18)	14 (25.9)	*0.331*
Systemic hypertension, n (%)	54 (51.9)	28 (56)	26 (48.1)	*0.423*
Congestive Heart Failure, n (%)	1 (0.9)	0 (0)	1 (1.8)	*0.334*
COPD, n (%)	5 (4.8)	2 (4)	3 (5.6)	*0.711*
CKD, n (%)	12 (11.5)	9 (18)	3 (5.6)	***0.047***
Hemodialysis, n (%)	7 (6.7)	5 (10)	2 (3.7)	*0.200*
Liver disease, n (%)	4 (3.8)	2 (4)	2 (3.7)	*0.937*
Solid tumor, n (%)	19 (18.3)	16 (32)	3 (5.6)	***0.0001***
Hematological malignancy, n (%)	5 (4.8)	1 (2)	4 (7.4)	*0.198*
Immunosuppressant therapy, n (%)	5 (4.8)	2 (4)	3 (5.6)	*0.711*
Obesity, n (%)	10 (9.6)	5 (10)	5 (9.3)	*0.898*
SAPS II, median (IQR)	38 (30–46)	40 (28–45)	36 (30–49)	*0.74*
Presence of central line, n (%)	90 (86.5)	43 (86)	47 (87)	*0.877*
Septic shock, n (%)	35 (33.6)	12 (24)	23 (42.6)	*0.05*
Mechanical ventilation*, n (%)	51 (49.5)	22 (44)	29 (53.7)	*0.323*
CRP, median (IQR), mg/dL	17.6 (8.5–34.1)	16.8 (8.7–34.1)	20 (7.4–42.5)	*0.603*
Procalcitonin, median (IQR), ng/dL	1.1 (0.3–3.9)	0.9 (0.3–7.6)	1.4 (0.3–3.3)	*0.777*
CRRT*, n (%)	11 (10.6)	3 (6)	8 (14.8)	*0.144*
ECMO*, n (%)	5 (4.8)	1 (2)	4 (7.4)	*0.198*
Source of infection: skin and soft tissue, n (%)	1 (1)	1 (2)	0 (0)	*0.296*
Source of infection: IAI, n (%)	0 (0)	0 (0)	0 (0)	*NA*
Source of infection: HAP, n (%)	10 (9.6)	4 (8)	6 (11.1)	*0.591*
Source of infection: VAP, n (%)	27 (26)	14 (28)	13 (24.1)	*0.648*
Source of infection: catheter-related, n (%)	21 (20.2)	13 (26)	8 (14.8)	*0.156*
Primary BSI, n (%)	45 (43.3)	18 (36)	27 (50)	*0.150*
Polymicrobial BSI	23 (22.1)	9 (18)	14 (25.9)	*0.331*
Early appropriate antibiotic treatment, n (%)	61 (59.2)	32 (64)	29 (54.7)	*0.338*
Time to definite therapy, median (IQR), days	1 (0–3)	1 (0–2)	1 (0–2)	*0.770*
Appropriate definite therapy within 48 h, n (%)	74 (71.1)	38 (76)	36 (66.7)	*0.294*
Monotherapy	26 (25)	14 (28)	12 (22.2)	*0.759*
Combination therapy, n (%) 1. FOF 2. MEM 3. TGC 4. A/S	78 (75) 32 (30.8) 8 (7.7) 12 (11.5) 42 (40.4)	36 (72) 12 (24) 1 (2) 6 (12) 24 (48)	42 (77.7) 20 (37) 7 (12.9) 6 (11.1) 18 (33.3)	*0.759* *0.150* ***0.036*** *0.887* *0.128*
Source control (when indicated), n (%)	16/22 (63.6)	8/14 (57.1)	6/8 (75)	*0.187*
CFDC susceptibility, n (%)	59 (56.3)	26 (52)	33 (61.1)	*0.349*
Early clinical improvement, n (%)	57 (54.8)	32 (64)	25 (46.3)	*0.070*
Clinical cure, n (%)	57 (54.8)	33 (66)	24 (44.4)	***0.027***
Microbiological eradication, n (%)	71 (68.9)	36 (72)	35 (66)	*0.513*
Relapse after clinical cure, n (%)	9 (8.6)	4 (8)	5 (9.3)	*0.819*
Superinfection, n (%)	36 (34.6)	16 (32)	20 (37)	*0.591*
Adverse events, n (%)	26 (25)	5 (10)	21 (38.8)	***0.001***
AKI, n (%)	21 (20.2)	0 (0)	21 (38.9)	***< 0.0001***
7-day mortality, n (%)	19 (18.3)	8 (16)	11 (20.4)	*0.564*
14-day mortality, n (%)	28 (26.9)	11 (22)	17 (31.5)	*0.276*
30-day mortality, n (%)	41 (39.4)	18 (36)	23 (42.6)	*0.492*
Overall mortality, n (%)	61 (59.2)	28 (57.1)	33 (61.1)	*0.682*
Length of hospital stay, median (IQR), days	47.5 (28.5–85.5)	57 (30–87)	44 (27–75)	*0.273*
Length of ICU stay, median (IQR), days	34 (20–67)	36.5 (18.5–76)	31 (22–53)	*0.415*

CFD: cefiderocol; COL: colistin; ICU: Intensive Care Unit; CCI: Charlson Comorbidity Index; COPD: Chronic Obstructive Pulmunary Disease; CKD: Chronic Kidney Disease; CRP: C-reactive protein; CRRT: Continuous Renal Replacement Therapy; ECMO: ExtraCorporeal Membrane Oxygenation; IAI: intra-abdominal infection; HAP: hospital-acquired pneumonia; VAP: ventilator-associated pneumonia; BSI: bloodstream infection; FOF: Fosfomycin; MEM: meropenem; TGC: tigecycline; A/S: ampicillin/sulbactam. AKI: Acute kidney injury. *: at the time of infection

Combination therapy was administrated in the majority of patients (78, 75%), of which 36 (72%) treated with CFDC regimens and 42 (77.7%) with COL regimens. The most common associated antibiotic was A/S [median dosage 24 gr/die (range 6–27)], used overall in 42 (40.4%) patients [24 (48%) vs. 18 (33.3%) in CFDC and COL groups, respectively], followed by FOF in 32 (30.8%) patients [median dosage 16 gr/die (range 6–18)] [12 (24%) vs. 20 (37%) in CFDC group and COL group, respectively]. Meropenem was mainly combined with COL than CFDC (12.9% vs. 2%, *p* = 0.036). All the regimens are described in Supplementary Fig. 1.

### Outcomes

**Fig. 1:**
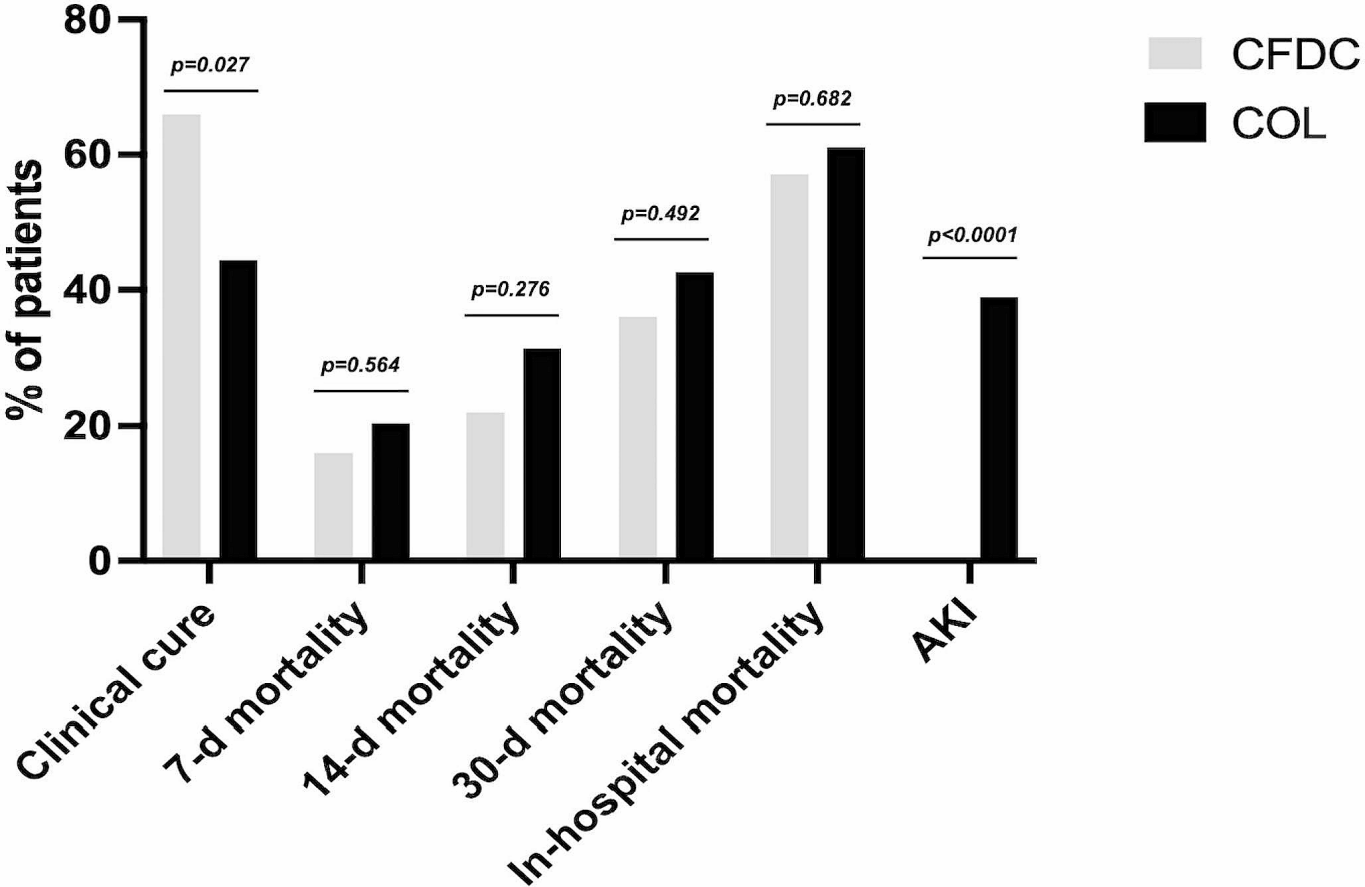
Study outcomes and adverse events according to CFDC or COL regimens. CFDC: cefiderocol; COL: colistin. AKI: Acute Kidney Injury

While the 7-day, 14-day, and 30-day mortality rates did not show significant differences between the two groups, they were lower for the CFDC group compared to the COL group (16% vs. 20.4%, 22% vs. 31.5% and 36% vs. 42.6% for CFDC and COL, respectively). Notably, clinical cure was significantly higher in CFDC than in COL group (66% vs. 44.4%, *p* = 0.027) (Fig. 1). Details of the comparison between patients with and without clinical cure are shown in Supplementary Table 1.

**Fig. 2:**
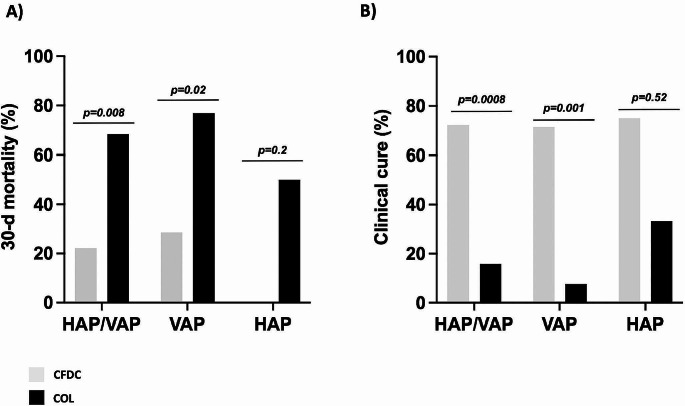
30-d mortality (Panel **A**) and clinical cure (Panel **B**) rates in patients receiving CFDC or COL regimens for HAP or VAP. CFDC: cefiderocol; COL: colistin. HAP: hospital-acquired pneumonia; VAP: ventilator-associated pneumonia

After stratification according to the source of infection, we found that patients with HAP/VAP treated with CFDC had a statistically significant lower 30-d mortality and higher clinical cure than those treated with COL (22.2% vs. 68.4%, *p* = 0.008, and 72.2% vs. 15.8%, *p* = 0.0008, respectively), especially in patients with bacteremic VAP (28.6% vs. 76.9%, *p* = 0.02 and 71.4% vs. 7.7% *p* = 0.001, respectively) (Fig. 2, panel A-B).

Patients treated with COL exhibited a higher incidence of adverse events compared to those treated with CFDC regimens (38.8% vs. 10%, *p* < 0.0001), primarily attributed to acute kidney injury (AKI) in the COL group (Fig. 1). In detail, patients receiving CFDC experienced mild gastrointestinal toxicity (*n* = 4) and increase in sodium values (*n* = 1), the latter probably due to concomitant A/S administration, while AKI was observed in all the patients treated with COL.

No differences between the two groups were observed as for microbiological cure and rates of infection recurrence or secondary infections (Table 1).

### Predictors of 30-d mortality

Comparison between 30-d survivors and non-survivors is shown in Table 2. A significative higher mortality was found in older patients (*p* = 0.040), those with SARS-CoV2 infection (*p* = 0.002), higher CCI (*p* = 0.0019), septic shock (*p* < 0.001), CRRT (*p* = 0.017) and ECMO (*p* = 0.004), while source control (when indicated) was protective (*p* = 0.028), as well as early clinical improvement and clinical cure (*p* < 0.0001 each).

**Table 2:** Comparison of 30-d survivors and non-survivors

	30-d survivors n (%) = 63 (60.6)	30-days non survivors n (%) = 41 (39.4)	*p*-value
Male sex, n (%)	40 (63.5)	31 (75.6)	*0.194*
Age, median (IQR), years	65 (56–76)	68 (61–81)	***0.040***
ICU stay, n (%)	36 (57.1)	30 (73.2)	*0.097*
SARS-CoV-2 co-infection, n (%)	19 (30.2)	25 (60.9)	***0.002***
Charlson Comorbidity Index, median (IQR)	4 (2–5)	6 (3–8)	***0.0019***
CCI ≥ 3, n (%)	43 (68.2)	33 (80.5)	*0.169*
Diabetes, n (%)	9 (14.3)	14 (34.1)	***0.017***
Systemic hypertension, n (%)	31 (49.2)	23 (56.1)	*0.492*
COPD, n (%)	2 (3.2)	3 (7.3)	*0.335*
CKD, n (%)	5 (7.9)	7 (17.1)	*0.154*
Hemodialysis, n (%)	2 (3.2)	5 (12.2)	*0.073*
Liver disease, n (%)	3 (4.8)	1 (2.4)	*0.547*
Solid tumor, n (%)	11 (17.5)	8 (19.5)	*0.677*
Immunosuppressant therapy, n (%)	3 (4.8)	2 (4.9)	*0.978*
Obesity, n (%)	4 (6.3)	6 (14.6)	*0.161*
SAPS II, median (IQR)	33 (28–42)	44 (34–47)	*0.104*
Septic shock, n (%)	13 (20.6)	22 (53.7)	***< 0.001***
Mechanical ventilation*, n (%)	23 (36.5)	28 (68.3)	***0.002***
CRP, median (IQR), mg/dL	14.9 (4.4–36.7)	21.7 (12.9–44.8)	*0.019*
Procalcitonin, median (IQR), ng/dL	0.9 (0.2–3.7)	1.4 (0.5–4.2)	*0.322*
CRRT*, n (%)	3 (4.8)	8 (19.5)	***0.017***
ECMO*, n (%)	0 (0)	5 (12.2)	***0.004***
Source of infection: skin and soft tissue, n (%)	1 (1.6)	0 (0)	*0.418*
Source of infection: HAP, n (%)	7 (11.1)	3 (7.3)	*0.521*
Source of infection: VAP, n (%)	13 (20.6)	14 (34.1)	*0.125*
Source of infection: LRTI, n (%)	20 (31.7)	17 (41.5)	*0.312*
Source of infection: catheter-related, n (%)	16 (25.4)	5 (12.2)	*0.101*
Primary BSI, n (%)	26 (41.3)	19 (46.3)	*0.610*
Polymicrobial BSI	14 (22.2)	9 (21.9)	*0.974*
Early appropriate treatment, n (%)	38 (61.3)	23 (56.1)	*0.600*
Time to definite therapy, median (IQR), days	0 (0–3)	1 (0–3)	*0.571*
Definitive therapy within 48 h, n (%)	45 (71.4)	29 (70.3)	*0.939*
Combination therapy, n (%) 1. FOF 2. MEM 3. TGC 4. A/S	48 (76.2) 18 (28.6) 6 (7.9) 9 (14.3) 26 (41.3)	30 (73.1) 14 (34.1) 3 (7.3) 3 (7.3) 16 (39)	*0.728* *0.547* *0.908* *0.277* *0.820*
Source control (when indicated), n (%)	13/17 (76.5)	1/5 (25)	***0.028***
Early clinical improvement, n (%)	49 (77.8)	8 (19.5)	***< 0.0001***
Microbiological eradication, n (%)	57 (91.9)	14 (34.1)	***< 0.0001***
Clinical cure, n (%)	54 (85.7)	3 (7.3)	***< 0.0001***
Length of hospital stay, median (IQR), days	76 (51–125)	30 (20–42)	***< 0.0001***
Length of ICU stay, median (IQR), days	55 (34–88)	23 (18–31)	***< 0.0001***

ICU: Intensive Care Unit; CCI: Charlson Comorbidity Index; COPD: Chronic Obstructive Pulmonary Disease; CKD: Chronic Kidney Disease; CRP: C-reactive protein; CRRT: Continuous Replacement Therapy; ECMO: ExtraCorporeal Membrane Oxygenation; IAI: intra-abdominal infection; HAP: hospital-acquired pneumonia; VAP: ventilator-associated pneumonia; BSI: bloodstream infection; FOS: Fosfomycin; MEM: meropenem; TGC: tigecycline; A/S: ampicillin/sulbactam. *: at the time of infection

At multivariable Cox regression analysis, ICU stay (HR 2.74, 95% CI 1.13–6.65, *p* = 0.025), SARS-CoV2 infection (HR 2.61, 95% CI 1.31–5.19, *p* = 0.006), ECMO (HR 8.63, 95% CI 2.68–27.77, *p* < 0.0001) and CCI (each point increment, HR 1.17, 95% CI 1.05–1.32, *p* = 0.005) were independently associated with 30-d mortality.

Receiving CFDC was not associated with the primary outcome (HR 0.91, 95% CI 0.45–1.82, *p* = 0.798), and this finding was further supported by the IPTW analysis (HR 0.74, CI 0.35–1.55, *p* = 0.431) (Table 3). The standardized mean differences before and after the IPTW procedure for each variable potentially influencing the treatment are shown in Supplementary Table 1.

**Table 3:** Cox-regression multivariable analysis for 30-d mortality predictors

	HR (95%CI)	*p*-value
CFDC (vs. COL)	0.91 (0.45–1.82)	*0.798*
CCI (each point increment)	1.17 (1.05–1.32)	***0.005***
ICU stay	2.74 (1.13–6.65)	***0.025***
SARS-CoV-2 co-infection	2.61 (1.31–5.19)	***0.006***
Septic shock	1.88 (0.89-4.00)	*0.097*
ECMO	8.63 (2.68–27.77)	***< 0.0001***
VAP (vs. other source of BSI)	0.77 (0.32–1.86)	*0.572*
Early appropriate antibiotic treatment	0.74 (0.37–1.48)	*0.407*
CRRT	0.76 (0.29–1.95)	*0.572*
**Inverse-probability treatment weight (IPTW)**		
CFDC (vs. COL)	0.74 (0.35–1.55)	0.431

CFDC: cefiderocol; COL: colistin; ICU: Intensive Care Unit; CCI: Charlson Comorbidity Index; ECMO: ExtraCorporeal Membrane Oxygenation; VAP: ventilator-associated pneumonia; BSI: bloodstream infection

### Microbiology analyses

CFDC susceptibility was available in 59 patients (56.3%), equally distributed between the groups (26/50, 52% and 33/54, 61.1% in CFDC and COL, respectively). CFDC was *in-vitro* susceptible in all but one subjects, with MIC values ranging from 0.094 to 1.5 µg/mL. The patient with CFDC resistance even before CFDC therapy had MIC 4 µg/mL, exhibited also COL resistance and was eventually treated with COL, MEM and A/S.

Emergence of CFDC in vivo resistance was observed in one patient (MIC 4 µg/mL). Unfortunately, we could not estimate the actual MIC before CFDC treatment since only disk diffusion was available.

COL resistance was observed in 13/104 patients (12.5%). All the strains were resistant to A/S, with MIC > 16/8 µg/mL.

### Use of CFDC

CFDC was mostly used in combination (36/50, 72%), particularly with A/S (48%) and FOF (24%). CFDC adjustment for renal function was noted in 12 patients, with a slightly higher 30-day mortality observed in those receiving CFDC adjusted for renal function (7/18, 38.8% vs. 5/32, 15.6%, *p* = 0.08), although the difference did not reach statistical significance. In particular, the patient who experienced in vivo resistance to CFDC was obese and on hemodialysis while treated.

## Discussion

In this study, we demonstrated that, although the overall mortality was only slightly lower in the CFDC-treated patients, the clinical cure rate was significantly higher in the CFDC group compared to the COL group. More importantly, within the subgroup of patients with HAP/VAP, the administration of CFDC was associated with a statistically significant decrease in 30-day mortality and an increase in clinical cure compared to COL. Furthermore, CFDC was associated with a statistically significant lower rate of adverse events than COL, particularly in terms of renal failure.

The role of CFDC as a potential first therapeutic option in CRAB infections is still under debate. Although the CREDIBLE-CR study indicated higher mortality rates in the subgroup of CRAB-infected patients treated with CFDC [13], leading to current guidelines not endorsing its use [32–34], real-life experiences have been accumulating evidence supporting the potential benefit of CFDC over COL-based regimens, particularly in the context of BSI and lung infections [17, 19–22, 35].

Only one retrospective observational study including severe CRAB infections, of which 47.7% were bacteremic, reported a lower 30-d mortality in those treated with COL compared to CFDC-containing regimens [18].

Consistent with these findings, a recent systematic review demonstrated that, compared to alternative therapies (mostly colistin-based), patients treated with CFDC-based regimens had a lower risk of mortality when the analysis was focused on observational studies with adjustments for confounding factors [36].

In our study, we demonstrated that patients with HAP/VAP treated with CFDC had a statistically significant advantage in terms of mortality and clinical cure than those receiving COL, and this finding was even more evident in the setting of VAP. ELF penetration of antibiotics in critically ill patients with VAP remains still a concern. As a matter of fact, COL penetration in the ELF is poor [7], while, at standard dosing, CFDC exposure in the ELF is similar to that of other cephalosporins and has been demonstrated to achieve ELF concentrations sufficient for treating Gram-negative bacteria with a MIC of 4 mg/L [37]. Nevertheless, suboptimal PK/PD CFDC targets could occur, leading to microbiological failure [38].

We confirmed the role of SARS-CoV2 infection and ECMO as independent predictors of unfavorable outcome [19, 39–42], the latter probably due to a significantly reduced serum concentrations of specific antibiotics [43]. Furthermore, we found that the burden of comorbidities was independently associated with mortality. Indeed, our study population was extremely complex and frail, with a median CCI of 5 [44, 45], which was higher than that reported in many studies in the literature [19, 20] and may potentially explain the lack of statistically significant differences observed between the two groups.

These findings underscore that the presence of multiple comorbidities may have a crucial role in worsening treatment outcomes, even if antimicrobial agents have been appropriately and timely prescribed. In this context, CFDC undoubtedly exhibited a safer profile than COL, particularly concerning renal function, suggesting a net advantage in favor of CFDC for frail patients, where the risk of acute renal failure during therapy should be avoided.

Our results may also be influenced by the fact that in only 52% of patients receiving CFDC, drug susceptibility has been available. While all but one of the tested strains were susceptible to CFDC, we could not exclude that some other CRAB isolates may have been less susceptible, or even resistant, to CFDC. Indeed, resistance to CFDC in MDR Gram-negatives has emerged even before its widespread use in clinical practice [46–48]. In addition, development of resistance during treatment has also been observed, suggesting the need to test all isolates before and during CFDC administration [19]. Interestingly, heteroresistance is highly prevalent in CRAB, but its clinical impact is still unclear [49].

In line with the current literature [19, 20], we showed that CFDC was predominantly employed in combination, with A/S and FOF being the most frequently administered partner drugs. Despite the in vitro resistance of all our strains to A/S, the decision to still use this drug was based on the fact that, when given in high doses, such as ours, sulbactam has the capability to saturate PBP-1 and PBP-3 and may therefore overcome the increasing rates of sulbactam resistance [5]. Unfortunately, due to the low number of patients, we were not able to state whether a specific combination was associated with a better outcome, even though recent studies suggest a possible benefit for the combination CFDC plus FOF [17, 20].

Although CRAB infections are considered peculiar of the ICU, we showed that approximately one third of patients with CRAB BSI acquired the infection outside the critical care setting. This finding has important clinical implications and should raise the awareness that also patients not in the ICU may be at risk of CRAB infections, influencing not only the appropriateness of early therapy, which is a well-known predictor of survival, but also the infection control policies within the hospital [45].

Our study undoubtedly presents several limitations. First, it is a retrospective single center study, thus not leading to a generalization of the results, and the selection of antimicrobial therapies was based on the clinical judgement of physicians. Secondly, we could not obtain CFDC susceptibility in all the patients receiving the drug, and therefore we could not exclude with certainty that some patients may have had a CRAB infection sustained by a less susceptible strain, possibly influencing our results. Thirdly, we acknowledge that the two study populations exhibited distinct features that could have possibly influenced the choice of treatment and the outcome. However, we conducted the IPTW analysis to balance the covariates and to reduce potential bias related to the heterogenicity of population and the retrospective nature of the study.

Additionally, not all the infections were monomicrobial. Nevertheless, among HAP/VAP, only two were truly polymicrobial. Hence, we believe that the better outcome observed in HAP/VAP for CFDC than COL was not influenced by the presence of pathogens other than CRAB. Lastly, but not less important, we could not assess the serum CFDC concentrations in our study cohort, which may have given possible insights on the observed worse outcome in some conditions.

## Conclusion

In conclusion, our data suggest that CFDC could be an effective and safe treatment option for CRAB BSI, especially in patients with HAP/VAP as well as frail patients where the risk of acute renal failure during therapy should be avoided. In our real-life experience, CFDC was mostly used in combination therapy, either with A/S or FOF. Further investigations are needed to assess the exact role of CFDC for the treatment of CRAB infections.

### Electronic supplementary material

Below is the link to the electronic supplementary material.

Supplementary Material 1

Supplementary Material 2

Supplementary Material 3

Supplementary Material 4

## Data Availability

All data relevant to the study are included in the article and are available from the corresponding author upon request.

## Code availability

All statistical analyses were performed using STATA™ software, v. 17 (StataCorp) and Graphpad Prism™, charts using Microsoft Office™ and Graphpad Prism™.
