# Supplementary material for: Clinical effectiveness of cefiderocol for the treatment of bloodstream infections due to carbapenem-resistant Acinetobacter baumannii during the COVID-19 era: a single center, observational study
Source: Eur J Clin Microbiol Infect Dis. 2024 Apr 18;43(6):1149–60. doi: 10.1007/s10096-024-04833-8 (PMC11178648; doi:10.1007/s10096-024-04833-8)
Supplement: Supplementary file 1 — Supplementary Material 1 [file 10096_2024_4833_MOESM1_ESM.docx]

|  | **Clinical cure**  **n (%) = 57 (42.8)** | **Clinical failure**  **n (%) = 47 (45.2)** | ***p-values*** |
| --- | --- | --- | --- |
| **Male sex, n (%)** | 34 (59.6) | 37 (78.7) | ***0.038*** |
| **Cefiderocol use, n (%)** | 33 (57.9) | 17 (36.2) | ***0.027*** |
| **Age, median (IQR), years** | 67 (56-77) | 66 (60-79) | *0.268* |
| **ICU stay, n (%)** | 31 (54.4) | 35 (74.5) | ***0.034*** |
| **SARS-CoV-2 co-infection, n (%)** | 15 (26.3) | 29 (61.7) | ***<0.0001*** |
| **Charlson Comorbidity Index, median (IQR)** | 4 (2-5) | 5 (3-7) | *0.054* |
| **CCI≥3, n (%)** | 40 (70.2) | 36 (76.6) | *0.463* |
| **Diabetes, n (%)** | 8 (14) | 15 (31.9) | ***0.029*** |
| **Systemic hypertension, n (%)** | 33 (57.9) | 21 (44.7) | *0.179* |
| **Congestive Heart Failure, n (%)** | 0 (0) | 1 (2.1) | *0.268* |
| **COPD, n (%)** | 1 (1.7) | 4 (8.5) | *0.109* |
| **CKD, n (%)** | 5 (8.8) | 7 (14.9) | *0.331* |
| **Hemodialysis, n (%)** | 2 (3.5) | 5 (10.6) | *0.149* |
| **Liver disease, n (%)** | 3 (5.3) | 1 (2.1) | *0.408* |
| **Solid tumor, n (%)** | 11 (19.3) | 8 (17) | *0.765* |
| **Hematological malignancy, n (%)** | 1 (1.7) | 4 (8.5) | *0.109* |
| **Immunosuppressant therapy, n (%)** | 1 (1.7) | 4 (8.5) | *0.109* |
| **Obesity, n (%)** | 4 (7) | 6 (12.8) | *0.322* |
| **Septic shock, n (%)** | 12 (21) | 23 (48.9) | ***0.003*** |
| **Mechanical ventilation*, n (%)** | 20 (35.1) | 31 (65.9) | ***0.002*** |
| **CRP, median (IQR), mg/dL** | 14.9 (4.3-36.6) | 20.8 (12.8-43.4) | ***0.044*** |
| **Procalcitonin, median (IQR), ng/dL** | 1 (0.3-7.6) | 1.1 (0.3-3.3) | *0.627* |
| **CRRT*, n (%)** | 2 (3.5) | 9 (19.1) | ***0.010*** |
| **ECMO*, n (%)** | 1 (1.7) | 4 (8.5) | *0.109* |
| **Source of infection: skin and soft tissue, n (%)** | 1 (1.7) | 0 (0) | *0.362* |
| **Source of infection: HAP, n (%)** | 5 (8.8) | 5 (10.6) | *0.748* |
| **Source of infection: VAP, n (%)** | 11 (19.3) | 16 (34) | *0.088* |
| **Source of infection: catheter-related, n (%)** | 14 (24.6) | 7 (14.9) | *0.222* |
| **Source of infection: lung, n (%)** | 16 (28.1) | 21 (44.7) | *0.078* |
| **Primary BSI, n (%)** | 26 (45.6) | 19 (40.4) | *0.595* |
| **Early appropriate antimicrobial treatment, n (%)** | 36 (64.3) | 25 (53.2) | *0.254* |
| **Definite therapy within 48h, n (%)** | 42 (73.7) | 32 (68.1) | *0.530* |
| **Combination therapy, n (%)** | 46 (80.7) | 38 (80.5) | *0.985* |
| **Source control, n (%)** | 11/15 (73.3) | 3/7 (42.8) | *0.158* |
| **Acute kidney injury, n (%)** | 9 (15.8) | 13 (27.7) | *0.140* |
| **Early clinical improvement, n (%)** | 48 (84.2) | 9 (19.1) | ***<0.0001*** |
| **Microbiological eradication, n (%)** | 53 (94.6) | 18 (38.3) | ***<0.0001*** |
| **Relapse after clinical cure, n (%)** | 6 (10.5) | 3 (6.3) | *0.454* |
| **Superinfection, n (%)** | 22 (38.6) | 14 (29.8) | *0.347* |
| **Length of hospital stay, median (IQR), days** | 75 (44-116) | 32 (20-51) | ***<0.0001*** |
| **Length of ICU stay, median (IQR), days** | 45.5 (33-79) | 27 (18-37) | ***0.0003*** |

**Supplementary Table1.** Features of patients with CRAB BSI according to clinical cure or failure during therapy.

CRAB: carbapenem-resistant *Acinetobacter baumannii*; BSI: bloodstream infection; ICU: Intensive Care Unit; CCI: Charlson Comorbidity Index; COPD: Chronic Obstructive Pulmunary Disease; CKD: Chronic Kidney Disease; CRP: C-reactive protein; CRRT: Continuous Replacement Therapy; ECMO: ExtraCorporeal Membrane Oxygenation; IAI: intra-abdominal infection; HAP: hospital-acquired pneumonia; VAP: ventilator-associated pneumonia. *: at the time of infection.
