# Supplementary material for: Clinical effectiveness of cefiderocol for the treatment of bloodstream infections due to carbapenem-resistant Acinetobacter baumannii during the COVID-19 era: a single center, observational study
Source: Eur J Clin Microbiol Infect Dis. 2024 Apr 18;43(6):1149–60. doi: 10.1007/s10096-024-04833-8 (PMC11178648; doi:10.1007/s10096-024-04833-8)
Supplement: Supplementary file 2 — Supplementary Material 2 [file 10096_2024_4833_MOESM2_ESM.docx]

**Supplementary Table2.** Standardized mean difference before and after the IPTW procedure for each variable potentially influencing the treatment (p=0.95). CKD: Chronic Kidney Disease.

|  | **Raw**  **Standardized differences** | **Weighted**  **Standardized differences** | **Raw**  **Variance Ratio** | **Weighted**  **Variance Ratio** |
| --- | --- | --- | --- | --- |
| SARS-CoV-2 co-infection | - 0.471 | - 0.039 | 0.843 | 0.988 |
| CKD | 0.385 | 0.099 | 2.767 | 1.302 |
| Solid tumor | 0.708 | 0.077 | 4.079 | 1.148 |
| Septic shock | - 0.415 | - 0.0006 | 0.743 | 0.998 |
