# Supplementary figures and images for: Clinical effectiveness of cefiderocol for the treatment of bloodstream infections due to carbapenem-resistant Acinetobacter baumannii during the COVID-19 era: a single center, observational study
Source: Eur J Clin Microbiol Infect Dis. 2024 Apr 18;43(6):1149–60. doi: 10.1007/s10096-024-04833-8 (PMC11178648; doi:10.1007/s10096-024-04833-8)

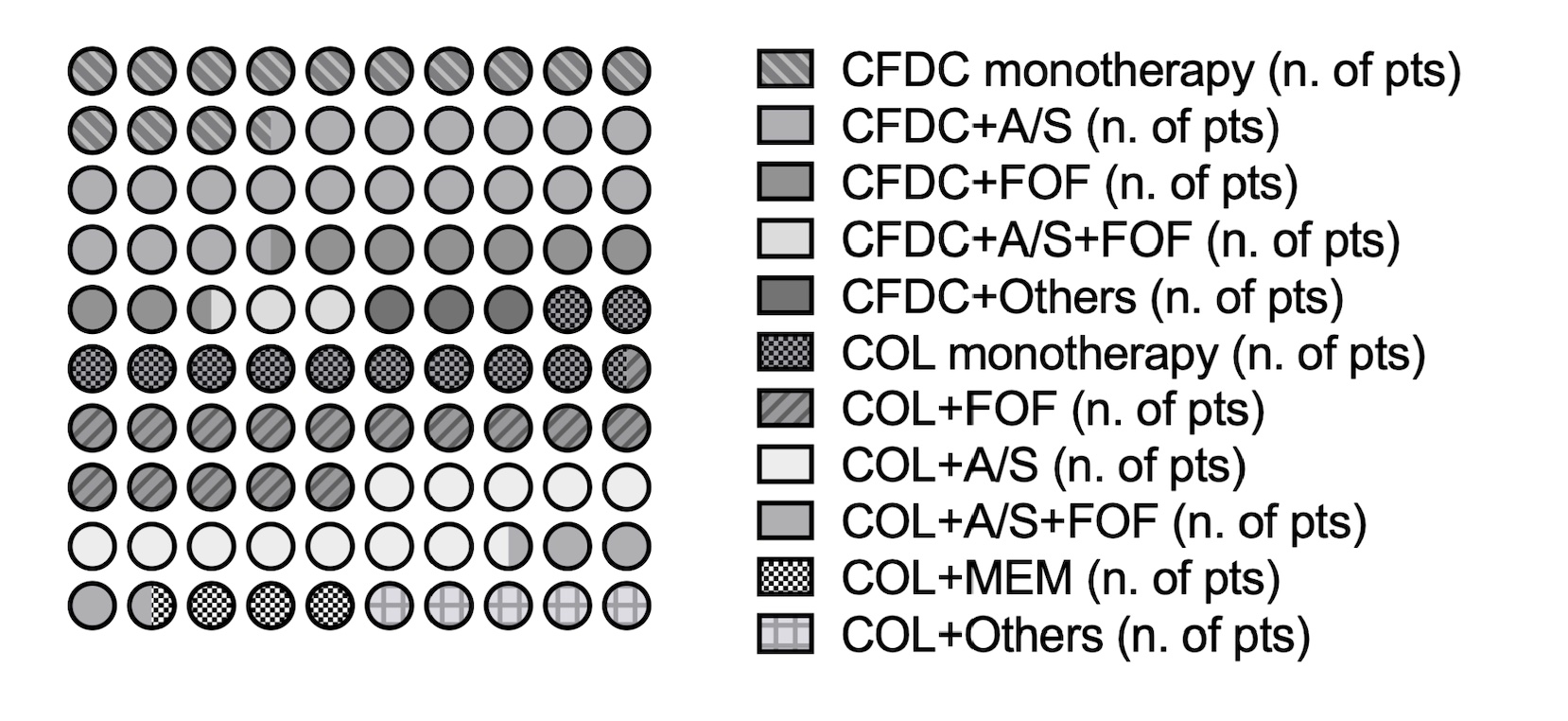

Supplement: Supplementary file 3 — Supplementary Material 3 [file 10096_2024_4833_MOESM3_ESM.jpg]
