# Supplementary material for: Clinical effectiveness of cefiderocol for the treatment of bloodstream infections due to carbapenem-resistant Acinetobacter baumannii during the COVID-19 era: a single center, observational study
Source: Eur J Clin Microbiol Infect Dis. 2024 Apr 18;43(6):1149–60. doi: 10.1007/s10096-024-04833-8 (PMC11178648; doi:10.1007/s10096-024-04833-8)
Supplement: Supplementary file 4 — Supplementary Material 4 [file 10096_2024_4833_MOESM4_ESM.docx]

**Supplementary materials**

**Supplementary Figure1.** Treatment regimens in the 104 patients with CRAB bloodstream infections. CRAB: carbapenem-resistant *A. baumannii*.

**Supplementary Table1.** Features of patients with CRAB bloodstream infections according to clinical cure or failure during therapy. CRAB: carbapenem-resistant *A. baumannii.*

**Supplementary Table2***.* Standardized mean difference before and after the IPTW procedure for each variable potentially influencing the treatment (p=0.95). CKD: Chronic Kidney Disease.
